# A multi-source data-driven framework for probabilistic flood risk assessment using cascade machine learning models: case study in the Sichuan Basin

**DOI:** 10.1038/s41598-025-12391-y

**Published:** 2025-07-23

**Authors:** Yan Lu, Ying Huang, Xiaoling Liu

**Affiliations:** 1 Research Center for Pacific Island Countries, Liaocheng University, Liaocheng, 252000 Shandong China; 2 China-Pacific Island Countries Climate Action Cooperation Center, Liaocheng University, Liaocheng, 252000 Shandong China; 3 Guangxi Academy of Sciences, Nanning, 530000 Guangxi China

**Keywords:** Flood frequency analysis, Physics-informed sequential modeling framework, Climate change, SVM-based machine learning model, Sichuan basin, Climate change, Hydrology

## Abstract

Along with global climate change, more frequent extreme climate phenomena have led to an increasing number of and increasingly severe flood disasters. Extreme rainfall events are capable of generating substantial amounts of surface runoff. Concurrently, the progression of urbanization has given rise to the expansion of impervious surfaces, thereby augmenting the likelihood of flood disasters. As China’s most flood-vulnerable region, the Sichuan Basin has sustained recurrent catastrophic flooding throughout history. This study establishes three policy-relevant hotspots across the basin as critical testbeds to quantify climate-driven changes in flood recurrence intervals. Utilizing CMIP6 projections under different SSPs scenarios, we developed a physics-informed process-based modeling framework integrating statistical downscaling, adjustments for extreme values and flood frequency analysis, specifically addressing how anthropogenically modified hydrological regimes amplify extreme event probabilities in this monsoon-dominated basin. The findings suggest that by the conclusion of the current century, the study area is likely to experience a notable increase in temperature, with an anticipated rise of approximately 1.7 ℃, and an intensification in precipitation, with an increase of 9.4% percent. Furthermore, the likelihood of extreme flood disaster events is projected to double, underscoring the imperative for robust climate adaptation and disaster mitigation strategies.

## Introduction

Flood disasters, as one of the natural disasters that have the greatest impact on human society, have become more frequent and have a more serious impact with the influence of global climate change^1,2^. Based on the report of the Intergovernmental Panel on Climate Change (IPCC) AR6 Synthesis Report: Climate Change 2023, for every 0.5 °C increase, extreme high temperatures, extreme precipitation and regional droughts will occur more frequently and with greater severity. Meanwhile, this will further lead to more serious secondary disasters, such as the flooding and urban waterlogging^3,4^. The extreme precipitation is one of the major cause of the flooding disaster^5^, it also presents a tendency to be short in duration but heavy in volume. The Sichuan Basin has become one of the regions most severely affected by flood disasters in China due to several factors: its high and concentrated rainfall, complex topography with an overall elevation higher in the west and lower in the east, numerous rivers with large water convergence, and the rapid increase in impermeable surfaces resulting from urbanization^6,7^. For example, the catastrophic flooding that occurred in the upper and middle reaches of the Yangtze River in 1998 resulted in severe breaches of levees, claimed over 2000 lives, and incurred economic losses exceeding US$25 billion^8^. Since the advent of the twentieth century, studies investigations have revealed that the moderate rainfall levels in the southwestern region of China have exhibited a diminishing tendency. Concurrently, the likelihood of heavy rainfall episodes has been persistently on the rise^9^. This dual trend has consequentially given rise to a notably elevated occurrence probability of severe flood disasters, signifying a concerning shift in the region’s hydrological patterns with potential implications for ecological stability, infrastructure integrity, and human livelihoods.

Surface runoff is the immediate precursor to the development of flood events^10^. Surface runoff—the flow of water over land resulting from precipitation or snowmelt—serves as an integrative indicator of how regional surface characteristics and land use practices influence flood generation^11^. The swift aggregation of substantial rainfall on the ground surface culminates in the emergence of a robust flow, termed surface runoff. Given the strong connection between rainfall patterns and surface runoff generation, precise modeling of meteorological parameters—especially precipitation and temperature—is essential for climate impact studies. Therefore, this investigation on climate change effects on flood probabilities emphasizes surface runoff simulation as a fundamental research focus^12,13^. This objective is accomplished by utilizing a framework that integrates Global Climate Model (GCM) data with meteorological data to model surface runoff. Furthermore, this framework is enhanced by incorporating the frequency of historically significant flood events, enabling a comprehensive assessment of the risk associated with flood occurrence.

Data-driven models for flood hazard prediction under climate change typically adopt an atmospheric-hydrological coupling framework. This approach first obtains meteorological outputs (e.g., precipitation, temperature) from atmospheric models, which are then fed into hydrological models. Consequently, reconciling spatiotemporal scale mismatches between these two model types becomes critical. Spatiotemporal downscaling methods effectively bridge atmospheric and hydrological models, primarily categorized into: statistical downscaling^14^, dynamic downscaling^15,16^, hybrid statistical-dynamic frameworks^17^. Notably, Tran et al.^18^ applied Long Short-Term Memory (LSTM) networks to downscale precipitation projections from five Global Climate Models (GCMs) under two Representative Concentration Pathway (RCP) scenarios. LSTM effectively captures historical precipitation patterns while projecting future variations, particularly for extreme events. Similar applications include Sun et al.^19^. Compared to statistical methods, dynamic downscaling offers stronger interpretability through physical constraints. For example, Tang et al.^20^ employed the Weather Research and Forecasting (WRF) model to simulate high-resolution (4-km, 1-h) extreme rainfall over eastern China. Their projections for 2070–2099 under RCP2.6, RCP4.5, and RCP8.5 scenarios revealed: skillful reproduction of mean precipitation and extreme hourly rainfall, increased intensity and frequency of extreme events across eastern China by 2100,maximum intensification under RCP8.5, indicating heightened flood risks with accelerated warming.

Integrating meteorological variables with hydrological models through scale conversion can utilize diverse coupling architectures. The conventional approach employs physics-based atmospheric models (e.g., WRF, RegCM) coupled with distributed/semi-distributed hydrological models (e.g., HEC-RAS^21,22^, SWMM^23^, MIKE FLOOD^24,25^) to establish foundational flood projection frameworks^26–28^. For instance, Jiang et al.^29^ coupled WRF with the SWAT model to simulate hydrological processes in mountainous watersheds. Here, WRF-generated rainfall inputs fed into SWAT revealed significant underestimation of monthly runoff during high-flow periods. This limitation was mitigated by implementing area-weighting methods and enhancing precipitation allocation mechanisms within SWAT’s subroutines, substantially improving model performance. Jam-Jalloh et al.^30^ further evaluated two coupling schemes: the distributed WRF-Hydro system versus the semi-distributed HEC-HMS model driven by WRF outputs. Through four rainfall event case studies in North China’s mountains, WRF-HEC-HMS outperformed for long-duration storms, while WRF-Hydro excelled in simulating short-duration floods with higher peak discharges. Similar comparative studies include Goodarzi et al.^31^.

The ongoing advancement of machine learning has enabled deep learning models to effectively capture complex nonlinear relationships, facilitating their integration into atmospheric-hydrological coupling systems for runoff and flood process simulation. Notably, Ding et al.^32^ developed a Spatio-Temporal Attention Long Short-Term Memory model (STA-LSTM) for flood event modeling across three Chinese basins. Benchmarking against conventional models—including Convolutional Neural Networks (CNN), Graph Convolutional Networks (GCN), standard LSTM, Spatial Attention LSTM (SA-LSTM), and Temporal Attention LSTM (TA-LSTM)—revealed STA-LSTM’s superior performance. Additionally, Fang et al.^33^ employed a Local Spatial Sequential LSTM (LSS-LSTM) for flood forecasting in Shangyou County, China, demonstrating enhanced capability in processing flood-associated spatial relationships through localized information capture. Yin et al.^34^ further innovated by implementing two transformer-based architectures for rainfall-runoff modeling under limited observational data constraints, achieving higher predictive accuracy than LSTM-based frameworks.

Current atmospheric-hydrological frameworks extensively integrate diverse model types, including physics-based atmospheric models, statistical downscalers, distributed/semi-distributed hydrological models, and machine learning approaches. However, critical limitations persist: (1) Physics-based atmospheric and hydrological models require precise parameter calibration (e.g., high-resolution soil, vegetation, and terrain data), where data gaps directly degrade simulation accuracy; (2) High-performance computing resources are essential for coupled systems like WRF-Hydro, demanding exceptional computational power, memory capacity, storage throughput, and network bandwidth. These constraints are exacerbated in data-sparse regions—particularly across developing countries—where inadequate ground observations and immature remote sensing fusion techniques elevate input uncertainty^35^. Consequently, machine learning frameworks offer compelling advantages through reduced parametric demands and computational efficiency. Our flood probability prediction framework for monsoon-dominated basins enables low-cost early warning systems for mountainous communities and small urban centers, aligning with "Early Warnings for All" (United Nations Office for Disaster Risk Reduction, UNDRR) initiative by delivering accessible decision-support tools.

This study establishes a flood risk assessment framework structured as 'GCM → Meteorological Data → Surface Runoff → Flood Identification’. This design addresses the strong nonlinearity and predictability challenges inherent in flood data, which arise from complex interactions among rainfall, topography, and surface characteristics^36–38^. Given that surface runoff comprehensively reflects flood-triggering mechanisms influenced by these factors, it serves as a critical proxy for flood occurrence. Consequently, we integrate observed meteorological data (precipitation and temperature) with high-resolution runoff reanalysis data for holistic flood risk evaluation. To ensure precise representation of heterogeneous data types, two innovative models separately simulate meteorological and runoff variables. For extreme values, an adjustments technique optimizes simulation fidelity. Notably, monthly runoff data are employed because floods manifest as cumulative hydrological processes: the monthly scale better captures slow-variable states (e.g., antecedent soil moisture, groundwater storage, reservoir levels) requiring > 10-day rainfall saturation thresholds, whereas daily data introduce noise from isolated storms^39^. Furthermore, daily runoff is characterized by high dynamism, featuring pronounced seasonality, persistent trends, and abrupt changes triggered by rainfall events, alongside measurement-induced noise. These complex characteristics—including high nonlinearity, non-stationarity, and elevated variability—collectively impede the precise simulation of discontinuous runoff responses in machine learning models due to inherent difficulties in capturing such irregular, rapidly shifting behaviors^40^. Therefore, monthly aggregation filters high-frequency noise (e.g., dry–wet alternation), allowing models to prioritize climate-forcing signals without overfitting from excessive parameterization.

## Study area, data and methods

### Study area

**Fig. 1:**
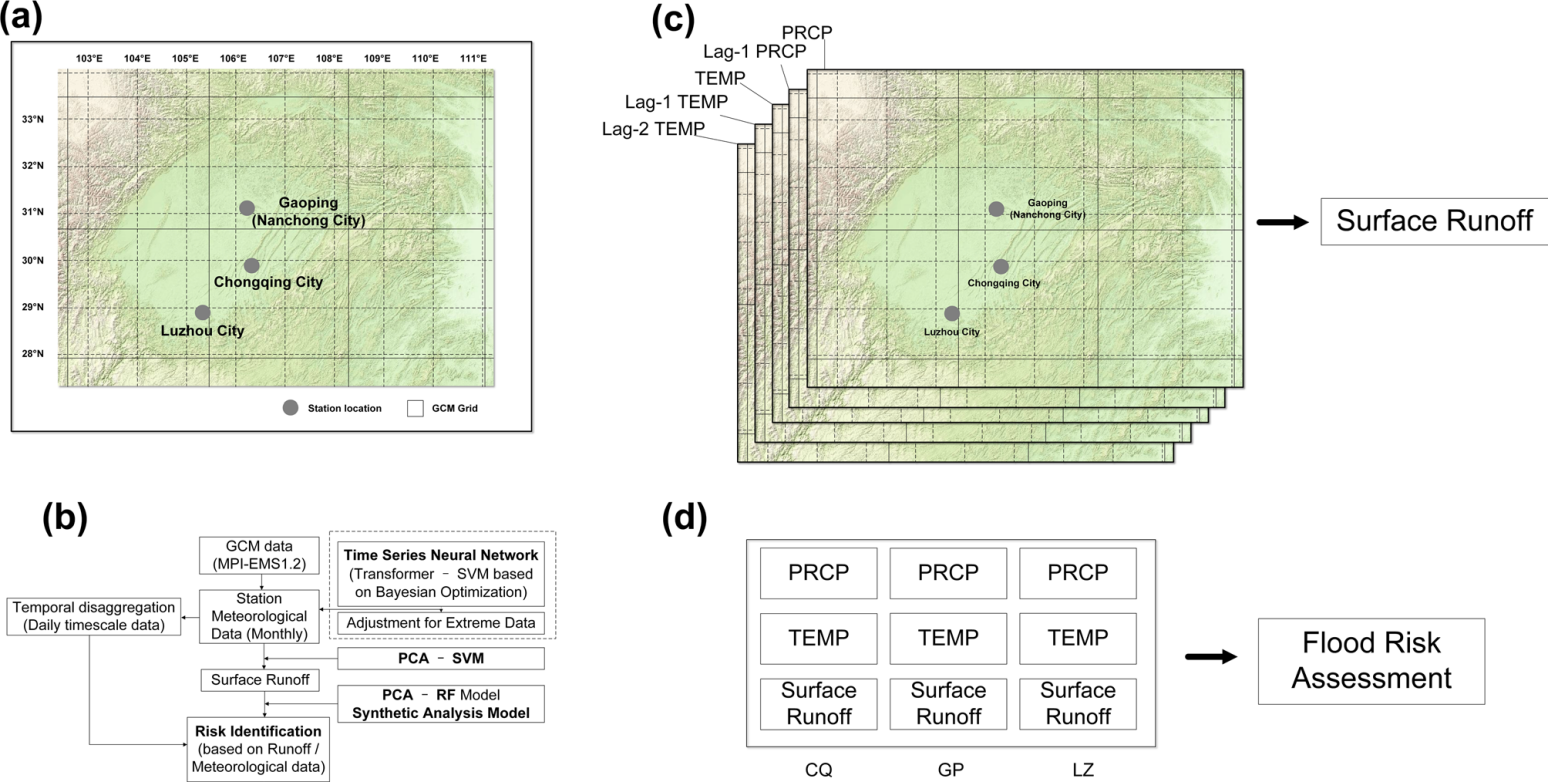
Location of the study area and meteorological stations, and methodology for flood risk assessment: (**a**) Geographic context, (**b**) analytical framework, (**c**) surface runoff input variables, (**d**) flood risk model input variables.

This study focuses on the Sichuan Basin, located in southwest of China, which is one of the regions most severely affected by flood disasters in the country. The Sichuan Basin is characterized by its complex topography, an intricate network of rivers, and frequent meteorological hazards. In particular, flood disasters resulting from torrential and exceptional rainfall events constitute one of the major meteorological calamities in the region^41^. In this study, three locations were selected as primary observation sites: Gaoping District (the abbreviation is “GP”, Nanchong City), Chongqing City (Municipality, the abbreviation is “CQ”), and Luzhou City (the abbreviation is “LZ”), which are situated in the northeastern, eastern and southern parts of the Sichuan Basin, respectively. The geographical distribution of the study region, along with the associated meteorological and hydrological observation stations (including gridded reanalysis runoff data), and GCM grid configurations are comprehensively illustrated in Fig. 1a.

### Datasets

In terms of datasets, the output data from the Max Planck Institute for Meteorology Earth System Model version 1.2 (MPI-ESM1.2^42,43^; was selected as the GCM data. Four socio-economic pathways (SSP1-2.6 with low radiative forcing, SSP2-4.5 as intermediate scenario, SSP3-7.0 representing high emissions, and SSP5-8.5 under very high forcing) were applied to evaluate flood disaster impacts under divergent climate conditions within the study area. The three observation stations provided meteorological data on daily precipitation and daily temperature.

The Surface Runoff data utilized in this study is sourced from the China Natural Runoff Dataset (CNRD) provided by Gou et al.^44^ and Miao et al.^45^. This dataset constitutes a long-term, high-quality, and spatiotemporally continuous record of natural runoff, with a spatial resolution as 0.01° × 0.01°. As of the time of this manuscript preparation, the dataset is available on a monthly time scale, with daily-scale data to be provided subsequently. Based on the temporal distribution of the aforementioned multiple datasets, the following approach was adopted in this study for the Validation and prediction phases. During the Validation phase, the model was trained using data from 1990 to 2006, and validation was conducted using data from 2007 to 2014. In the prediction phase, to maximize the utilization of training data, the model was trained using data spanning from 1990 to 2014, and predictions were made for the period from 2015 to 2100.

Flood event data were obtained from the Global Flood Database (GFD^46^), a validated repository containing georeferenced records of flood chronology, geographic coordinates, spatial extent, affected population, and economic losses. For probabilistic flood risk assessment in the Sichuan Basin, major flood events meeting impact severity thresholds (1990–2014) were extracted through systematic screening of the GFD metadata.

## Methods

### Multi-source data-driven framework

This study is dedicated to developing a framework for flood disaster assessment that can be applied in data-scarce regions, aiming to evaluate the changes in the probability of major flooding events within the study area under climate change conditions. The framework involves constructing a coupled model that integrates GCMs (MPI-EMS1.2 employed in this study), station-based meteorological variables (precipitation and temperature), and surface runoff data. Ultimately, through comprehensive analysis of the overall meteorological and runoff data within the study area, significant flood events are identified. The proposed methodology framework is presented in the Fig. 1b. Firstly, a coupled model of Transformer and Support Vector Machine (SVM) is used to perform spatial downscaling on precipitation and temperature data. Then, the simulated monthly precipitation (PRCP) and average temperature (TEMP) are input into a Principal Component Analysis (PCA)- SVM model for surface runoff simulation. Considering the temporal lag and physical correlation, five variables, namely PRCP, Lag-1 PRCP, TEMP, Lag-1 TEMP, and Lag-2 TEMP (as shown in Fig. 1c, are adopted for surface runoff data. When predicting surface runoff, accounting for the time lag between precipitation and temperature is crucial. This lag arises from fundamental physical processes within the hydrological cycle and energy transfer mechanisms. Key factors include: (1) subsurface flow dynamics, such as delayed aquifer recharge and retention of water in deep soil layers, and (2) evapotranspiration (ET) regulating soil moisture content, where high temperatures persistently deplete soil water via potential evapotranspiration (PET).

Ultimately, all the precipitation, temperature, and runoff data from stations within the study area are used as discriminant variables and input into a Synthetic Minority Over-sampling Technique (SMOTE) coupled PCA-random forest model (as illustrated in Fig. 1d) to identify the occurrence of floods. Meanwhile, to further understand the changes in precipitation, which is the climate variable most closely related to floods in the study area, the research framework also employs a KNN model to disaggregate monthly precipitation data into daily precipitation data. This allows for an analysis of the trends in daily precipitation under future climate conditions.

It is important to note that while the overall model framework adopts a more established approach compared to simpler single-model architectures, it offers significant advantages. First, for each variable, the optimal model is selected through comparative evaluation (as demonstrated in Supplementary Materials Fig. S1), minimizing error propagation inherent to cascade model structures. Second, the modular design enables site-specific customization; components can be readily replaced to suit local conditions in new study areas. Finally, the framework allows for seamless integration of improved machine learning algorithms as they become available, ensuring ongoing performance enhancement.

The proposed framework is grounded in physical mechanisms, following a staged process from climatic variables to precipitation/temperature downscaling, then to surface runoff simulation, and finally flood analysis. This physically based modeling chain captures key drivers: large-scale circulation → local temperature/precipitation → extreme hydrological events.

## Transformer- SVM

The Transformer-Support Vector Regression (Transformer-SVM) coupling model adopts a cascade architecture (see model schematic in Supplementary Materials Fig. S2) to leverage the strengths of both models for distinct processing stages. The Transformer serves as a powerful feature extractor: it derives high-level, context-aware feature representations from raw, complex data^37,47^. Key Transformer equations include:

1 $$Z = XW_{e} + P$$

2 $$head_{i} = Softmax\left( {\frac{{ZW_{i}^{Q} \left( {ZW_{i}^{K} } \right)^{T} }}{{\sqrt {d_{k} } }}} \right)ZW_{i}^{V}$$

3 $$T\_out = LayerNorm\left( {h_{{\left[ {CLS} \right]}} } \right)$$

where:

$X$: Input sequence matrix; $W_{e}$: Word embedding matrix; $P$: Positional encoding matrix; $Z$: Embedded input matrix; $W_{i}^{Q}$/$W_{i}^{K}$: *Query/Key* weight matrix for head $i$; $W_{i}^{V}$: Value weight matrix for head $i$; $d_{k}$: Attention head dimension; $h_{{\left[ {CLS} \right]}}$ : Final hidden state of the $\left[ {CLS} \right]$ token; $LayerNorm$: Layer Normalization; $T\_out$: Aggregated feature vector.

The SVM, recognized for its robustness and efficiency, utilizes the feature vector extracted by the Transformer as input to train a traditional SVM for regression^48,49^. The core SVM equation is:

4 $$f\left( Z \right) = \mathop \sum \limits_{i \in SV} \left( {\alpha_{i} - \alpha_{i}^{*} } \right)exp\left( { - \gamma T\_out_{i} - T\_out^{2} } \right) + b$$

where:

$\alpha_{i}$: Lagrange multiplier (≥ 0); $\gamma$ : Radial Basis Function (RBF) kernel parameter; $T\_out_{i}$: Feature vector of the $ith$ support vector (output from Transformer model); $b$: Bias term.

Equations above demonstrate that the cascaded Transformer-SVM model leverages the Transformer’s strength in feature extraction to capture long-range dependencies and complex contextual information within the input meteorological data. Simultaneously, the SVM component provides excellent generalization ability and robustness in high-dimensional feature spaces. SVMs typically perform well on small datasets, exhibit resistance to overfitting (especially with appropriate regularization parameters and kernel functions), and effectively handle nonlinear problems. Consequently, the Transformer-SVM model presents a highly competitive choice for scenarios involving limited data, constrained computational resources, or where model interpretability is desired. Furthermore, a Bayesian algorithm was employed to optimize the number of self-attention heads and the learning rate in the Transformer model. As detailed earlier, this model was applied to the spatial downscaling of precipitation and temperature data in this study. The model’s performance was compared against several traditional benchmarks, including standalone CNN, SVM, LSTM, Transformer models, and a PCA-SVM model for runoff simulation, demonstrating favorable results. This comparative analysis is presented in Fig. S1(a) and (b) of the Supplementary Materials.

### PCA-SVM

SVM, as established machine learning models, have been extensively applied in hydrology. Examples include river flow assessment^50^ and flood risk evaluation^51^. PCA is another classical algorithm as mentioned before in last , primarily used for exploratory data analysis and often integrated into hybrid predictive models^52^.

Given the high dimensionality, noise, and multicollinearity typical of hydrological data, this study employs PCA to reduce feature dimensions and remove noise. Key components extracted by PCA serve as inputs to a SVM model for prediction. This approach aims to enhance SVM’s performance and efficiency in high-dimensional regression tasks.

The PCA procedure involves:

1. Computing the covariance matrix of standardized training data,
2. Performing eigendecomposition to obtain eigenvalues and eigenvectors,
3. Determining the number of principal components based on a cumulative variance contribution threshold of 90% (validated via cross-validation).

By filtering low-variance (potentially noisy) and correlated redundant features, PCA enables SVM to learn more robust and generalizable regression functions, reducing sensitivity to irrelevant variables.

Model performance was compared against benchmarks including standalone CNN, SVM, LSTM, Transformer, and the proposed Transformer-SVM (see comparative results in Supplementary Materials Fig. S1(c)).

### SMOTE & PCA-RF hybrid model

Synthetic Minority Over-Sampling Technique (SMOTE) is a classic oversampling method specifically designed to address class imbalance problems in classification tasks^53^. Its core idea is to balance the dataset by generating synthetic minority class samples, thereby enhancing the model’s ability to recognize the minority class. In this study, the months associated with severe flood events constituted a minority of the samples within the overall time series (probability: 4.2%), posing a significant challenge to risk identification and classification due to class imbalance. To address this issue, the SMOTE algorithm was introduced to achieve balanced sample classification.

Consistent with its role in the PCA-SVM approach mentioned above, the PCA algorithm in the PCA-Random Forest model is primarily used for dimensionality reduction, selecting principal components with a high cumulative variance contribution rate. The Random Forest model enhances the accuracy and stability of flood identification by constructing multiple decision trees and aggregating their prediction results. Training the Random Forest model with data reduced by PCA can reduce computational load, improve training efficiency, and mitigate the risk of overfitting in the Random Forest model^54,55^.

### Adjustment for extreme values

Machine learning algorithms commonly exhibit underestimation bias in predicting extreme values, tending towards the mean. This bias primarily stems from two factors: (1) the use of loss functions such as MSE or RMSE, which heavily penalize large errors, incentivizing models to minimize overall MSE at the expense of accurately capturing extremes^56^ and (2) the inherent scarcity of data points in extreme-value regions, limiting the model’s ability to reliably learn the underlying patterns. However, accurately simulating extremes is crucial in this study for identifying extreme hazard events (e.g., heavy rainfall, heatwaves, floods). Therefore, a dedicated extreme-value adjustment procedure was implemented:

- During model training, the bias (difference between observed values and model outputs, e.g., from Transformer-SVM or PCA-SVM) was fitted to a Generalized Extreme Value (GEV) distribution to estimate its parameters (location $\mu$, scale $\sigma$, and shape $\xi$).
- Random bias samples (stochastic extreme-value bias) were then generated from this fitted GEV distribution (20 ensembles generated in this study).
- During prediction, these stochastic bias samples were added to the raw machine learning model outputs to produce the final ensemble predictions.

Evaluating the 20 ensemble members revealed that the ensemble mean performance closely resembled the raw model output. For instance, precipitation predictions at the LZ station showed the raw Transformer-SVM output had an RMSE of 63.41 mm. The ensemble mean after extreme-value adjustment was 64.94 mm, representing only a marginal 2.4% increase in RMSE. Crucially, however, the ensemble spread effectively encompassed extreme values, providing a more reliable basis for disaster risk assessment.

### Model evaluating criteria

In this study, the performance of the model is evaluated using the correlation coefficient ($R$), Root Mean Squared Error ($RMSE$)^49^, Nash–Sutcliffe efficiency coefficient ($NSE$)^33^^,^and percent bias ($PBIAS$)^57^ with the specific calculation methods outlined below. The model validation criteria required $NSE$ values exceeding 0.5 for monthly runoff simulations^58^, while $R$ surpassing 0.9 were maintained for monthly temperature reconstructions, consistent with hydrological modeling best practices. The $PBIAS$ evaluates systematic model bias through observed versus simulated data comparisons. Optimal agreement occurs at zero value, with positive $PBIAS$ indicating persistent underprediction and negative values demonstrating systematic overestimation of measured parameters. Model performance was classified as “Good” when the absolute value of PBIAS remained below 15%, following established hydrological model evaluation criteria^59^.

5 $${\text{R}} = { }\frac{{\mathop \sum \nolimits_{i = 1}^{n} \left( {OBS_{i} - \overline{OBS} } \right)\left( {SIM_{i} - \overline{SIM} } \right)}}{{\sqrt {\mathop \sum \nolimits_{i = 1}^{n} \left( {OBS_{i} - \overline{OBS} } \right)^{2} } \sqrt {\mathop \sum \nolimits_{i = 1}^{n} \left( {SIM_{i} - \overline{SIM} } \right)^{2} } }}{ }$$

6 $$RMSE = \sqrt {\frac{1}{n}\mathop \sum \limits_{i = 1}^{n} \left( {OBS_{i} - SIM_{i} } \right)^{2} }$$

7 $$NSE = 1 - \frac{{\mathop \sum \nolimits_{i = 1}^{n} \left( {OBS_{i} - SIM_{i} } \right)^{2} }}{{\mathop \sum \nolimits_{i = 1}^{n} \left( {OBS_{i} - \overline{OBS} } \right)^{2} }}$$

8 $$PBIAS = \mathop \sum \limits_{i = 1}^{n} \frac{{SIM_{i} - OBS_{i} }}{{OBS_{i} }} \times 100$$

where

$OBS_{i}$ =  $ith$ observed data. $SIM_{i}$ =  $ith$ simulated data. $\overline{OBS}$ =  Mean observed data. $\overline{SIM}$ =  Mean simulated data.

## Results

### Model validation

**Fig. 2:**
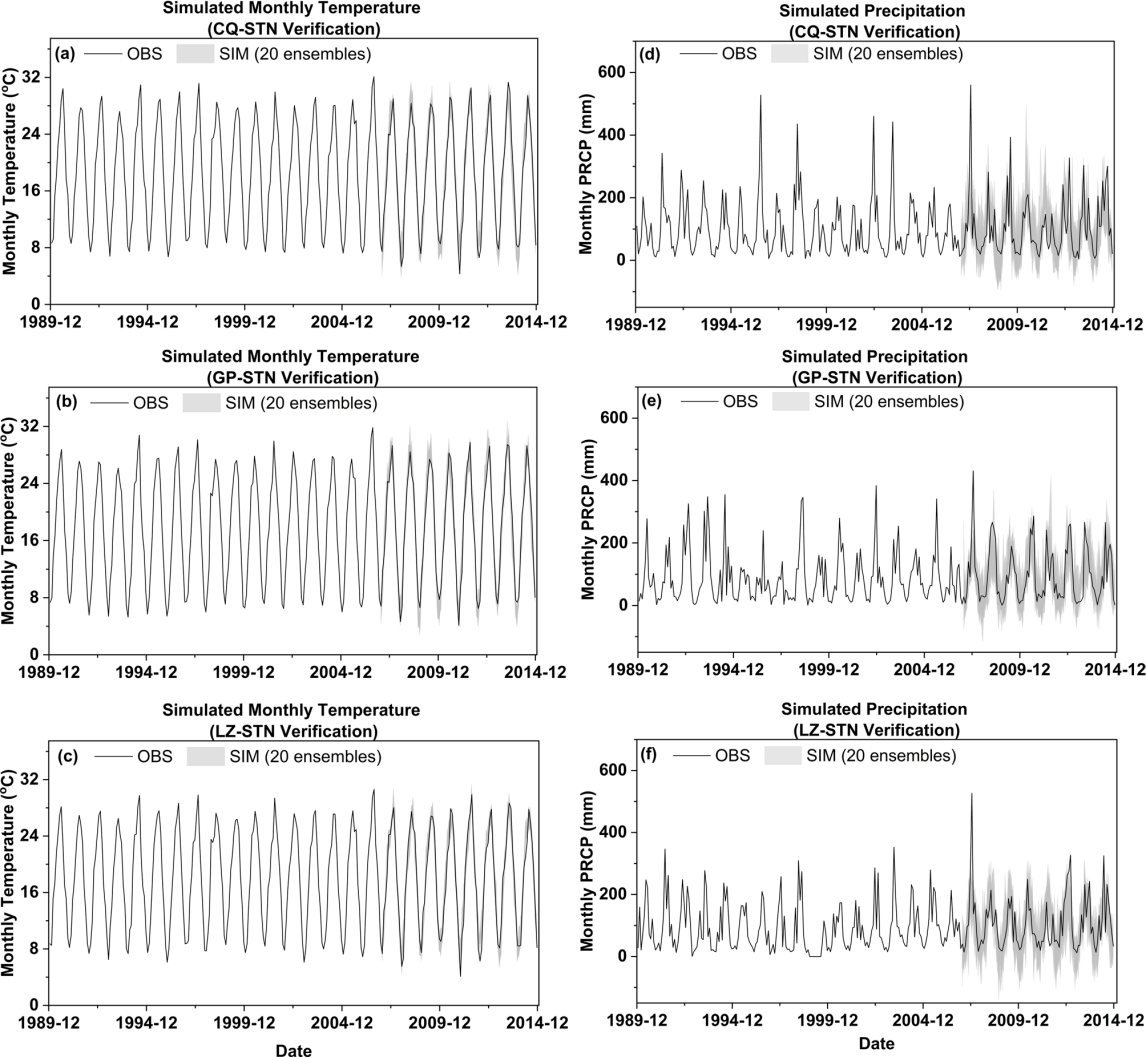
Validation of observed and simulated monthly mean temperature (**a–c**) and precipitation (**d–f**) across three stations (2007–2014): (**a**) Chongqing, (**b**) Gaoping, (**c**) Luzhou (temperature); (**d**) Chongqing, (e) Gaoping, (**f**) Luzhou (precipitation). Precipitation at Chongqing Station; (**e**) Monthly Precipitation at Gaoping Station; (**f**) Monthly Precipitation at Luzhou Station.

Figure 2 present the Validation of simulated monthly meteorological variables (average temperature and precipitation) from MPI-ESM1.2 through the Transformer-SVM with Bayesian optimization and extreme data generator. Figure 2a–c illustrates the observed and simulated monthly average temperature at three stations during the period 1990–2014. The simulation results during the validation period (2007–2014) demonstrate that the model effectively captures temperature variations. The model not only reproduces the temperature trends accurately but also encompasses the observed values within a narrow uncertainty range. Regarding extreme values, the maximum temperatures at the three stations during the validation period occurred in July 2013, reaching 31.3 °C, 29.5 °C, and 28.7 °C respectively. The minimum temperatures at these three stations were recorded in January 2011, at 4.3 °C, 4.1 °C, and 4.1 °C respectively. The simulated dataset effectively encompasses these observed extreme values. When comparing the average values of the simulated ensembles, the RMSE values for the three stations are 1.53, 1.46, and 1.65 respectively, while the correlation coefficients stand at 0.98, 0.98, and 0.97 respectively. This indicates that the model is capable of accurately reproducing the temperature metrics.

Figure 2d–f shows the comparison of observed and simulated monthly precipitation at 3 stations during the period of year 1990 to 2014. The simulation results for the three stations demonstrate that the model effectively captures the temporal sequence characteristics of monthly rainfall, especially during the latter half of the validation period. By comparing the observed values with the simulated means, the RMSE values for the three stations are76.5, 59.6, and 64.9, respectively. The PBIAS metrics at all three monitoring sites (Site A: − 10.65, Site B: − 10.25, Site C: − 11.5), consistently demonstrated negative values. This pattern indicates a relatively low cumulative bias in the simulations, yet reveals a systematic underestimation trend across the study area, aligning with Type II error characteristics defined in hydrological model validation frameworks^59^. The relatively larger RMSE deviations can be mainly attributed to two factors: (1) simulation biases resulting from the inherently strong randomness of rainfall,(2) the model’s difficulty in reproducing several extreme rainfall events during the validation period, such as the month of July 2007 when the monthly rainfall at all three stations close to or exceeded 500 mm. Due to the incorporation of the extreme data random regenerator and the presence of a small amount of data nearing or exceeding 400 mm in the training datasets from Chongqing and Gaoping observatories, a small portion of simulated data also exceeded 400 mm during the process of extreme value reproduction. However, there is a certain lag in the timing of their recurrence. Increasing the number of random ensembles could address such issues to some extent, but it would also result in a wider uncertainty range in the simulated dataset. The simulation results demonstrated that although the systematic underestimation bias in monthly precipitation modeling requires cautious interpretation regarding its hydrological impact on runoff/flood forecasting, the quantitative evaluation based on the PBIAS (absolute value < 25) indicates model performance satisfies the “Satisfactory” classification criteria established by Moriasi et al.^59^ for watershed-scale hydrological modeling.

**Fig. 3:**
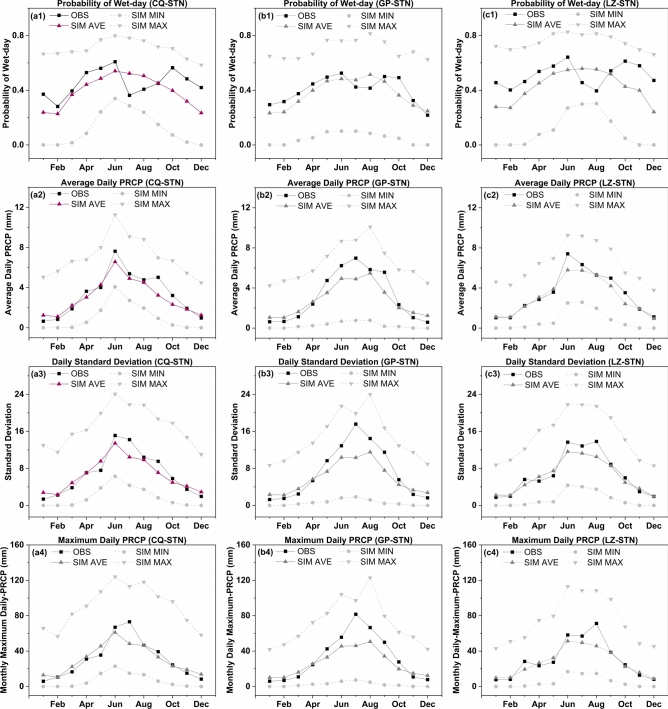
Validation of observed and simulated statistical characteristics ((1) Probability of Wet-day; (2) Average daily precipitation; (3) Standard deviation; (4) Maximum daily precipitation per month) of daily precipitation from 2007 to 2014 at three stations: (**a**) Chongqing Station; (**b**) Gaoping Station; (**c**) Luzhou Station.

**Fig. 4:**
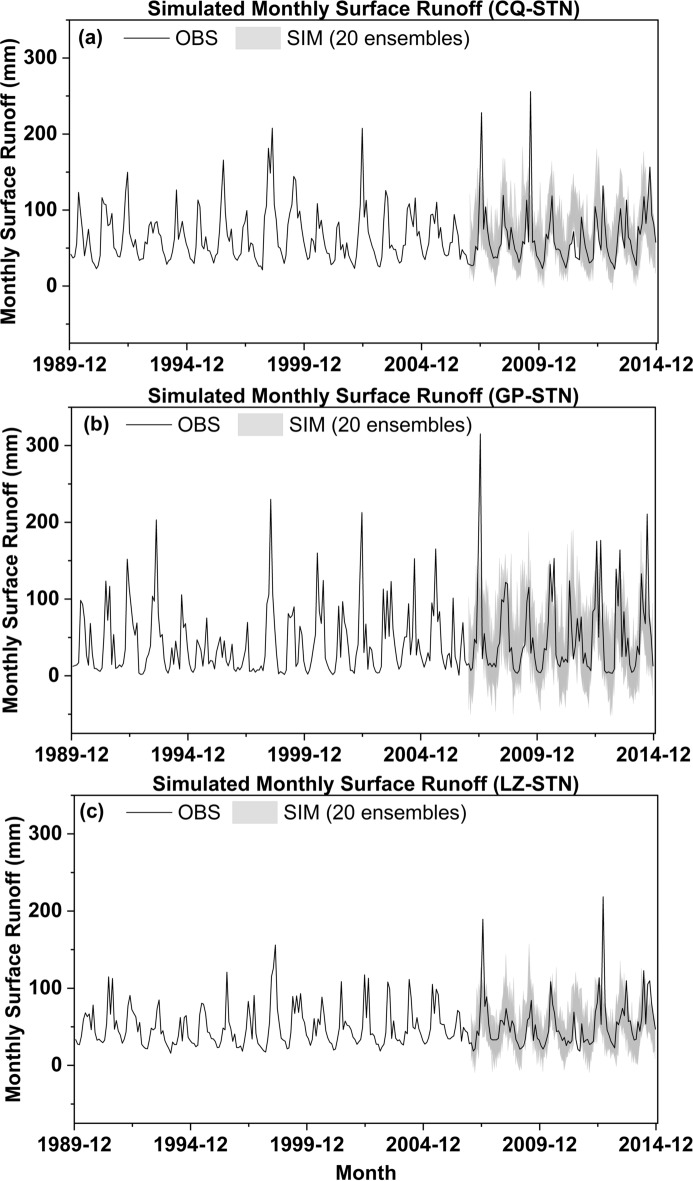
Validation of observed and simulated monthly surface runoff from 2007 to 2014 at Three Stations: (**a**) Chongqing Station; (**b**) Gaoping Station; (**c**) Luzhou Station.

As the precipitation is a crucial factor in generating runoff, which subsequently leads to flood disasters, and in order to provide a more precise description of model accuracy on a temporal scale, monthly rainfall data is decomposed into a daily timescale using the KNN method. Figure 3 illustrates four statistical attributes of daily precipitation at three stations, namely, the probability of wet days, the average daily precipitation amount, the standard deviation of daily precipitation, and the maximum daily precipitation value, respectively. By comparing the average values of various indicators (derived from 20 ensembles of monthly data), the simulated data generally reproduce the observed values quite well. For the indicator of probability of wet days (Fig. a1, b1 and c1), the RMSE values of three stations are 0.12, 0.06 and 0.14, respectively. But the simulation shows poor fit for the month of June and July, with similar issues observed at all three stations. The main reason for this issue is that the monthly rainfall data for June and July are relatively high, but the number of wet days is lower compared to adjacent months, exhibiting a certain degree of uniqueness. Additionally, the training dataset used for the KNN model did not distinguish between months in order to increase the training volume. Consequently, this led to biases in the indicators for June and July. This can be addressed in the future by training a separate model specifically for the unique months. The model-simulated statistical metrics of daily precipitation (mean and standard deviation) exhibited high consistency with observational datasets across the three monitoring stations, as illustrated in figures a2–c2 (mean values) and a3–c3 (standard deviations). Quantitative evaluation revealed strong agreement in both central tendency and variability: Correlation coefficients ($R$) for mean daily precipitation reached 0.96, 0.97, and 0.97 at stations CQ, GP, and LZ respectively ($RMSE$ values are 1.64, 1.04 and 0.65), while corresponding $R$ values for standard deviation demonstrated comparable performance (0.95, 0.97, and 0.97). Figures a4, b4, and c4 present the simulation results of the monthly maximum values. Compared to the observed values, the simulated values exhibit a slight underestimation, especially for the month of July. This situation is inherently related to the results of reproducing the monthly extreme values. Therefore, in this study, based on the simulated daily precipitation, the Gumbel distribution was used to calculate extreme rainfall values for different return periods. Table 1 presents the reproducibility results for three stations. The results indicate that the simulated values underestimate the observed data. This systematic deviation aligns with the demonstrated underestimation in monthly precipitation simulations (mean values) relative to observational datasets, as quantitatively validated through persistent negative PBIAS metrics (Fig. 4). Compared to the maximum values in the simulated ensembles, the underestimation rates for the 100-year flood event at the three stations are 5.7%, 2.6%, and 12.1%, respectively.

**Table 1:** Extreme precipitation quantile estimates corresponding to multi-return periods (T = 2–100 years) derived from stationary extreme value analysis at three stations.

Return period	CQ-STN			GP-STN			LZ-STN		
	OBS	Min	MAX	OBS	MIN	MAX	OBS	MIN	MAX
2 yr	96.95	75.98	114.76	105.62	60.22	101.42	88.97	73.65	105.76
3 yr	126.97	81.77	129.81	123.24	67.44	114.13	109.85	84.18	117.40
5 yr	151.86	86.58	150.57	137.86	73.42	129.46	127.16	92.88	127.06
10 yr	175.20	91.08	170.04	151.56	79.04	144.43	143.40	99.94	136.11
20 yr	192.35	94.39	184.34	161.63	83.16	155.42	155.33	105.13	142.76
30 yr	200.63	95.99	191.24	166.49	85.15	160.73	161.09	107.63	145.97
40 yr	205.92	97.01	195.65	169.60	86.43	164.12	164.77	109.23	148.03
50 yr	209.75	97.75	198.84	171.84	87.35	166.58	167.43	110.39	149.51
100 yr	220.38	99.80	207.71	178.09	89.90	173.39	174.83	113.61	153.63

To verify the simulation capability of the PCA-SVM coupled model for surface runoff, this section initially utilizes observed meteorological data, including precipitation and temperature, as input variables. The results, presented in Supporting Information Fig. S1 (c), demonstrate that the model effectively captures both the localized variations and the overall trend in surface runoff. However, it exhibits some underestimation of certain peak values. However, incorporating the downscaled temperature and precipitation data (20 ensemble members with extreme-value adjustment) yields modest improvement in simulating runoff extremes. Therefore, the PCA-SVM coupled model can effectively simulate surface runoff. Next, the study will use the simulated monthly meteorological data from the validation phase (2007–2014) of model framework as input conditions to verify whether the entire model framework can reproduce the surface runoff data.

Figure 4 shows the comparison of observed and simulated monthly surface runoff during the Validation period based on simulated meteorological data at three stations. The results indicate that the simulated values effectively capture the inter-annual variability of surface runoff data. However, due to the underestimation of a few extreme values in the simulated rainfall data, the runoff simulation data also exhibit a certain degree of underestimation bias in corresponding extreme cases. For instance, at the Chongqing station (Fig. 4a), two extreme events in July 2007 and August 2009 exceeded 220 mm, yet the maximum simulated values were only 120 mm and 144 mm, respectively. Although the simulated values suggest the possibility of extreme runoff during these months, the specific data are significantly underestimated. Similarly, at the Gaoping station (Fig. 4b), an extreme monthly runoff event in July 2007 exceeded 350 mm, but the maximum simulated value was only 153 mm. At the Luzhou station shown in Fig. 4c, while the average runoff data is lower than that of the other two stations mentioned, the extreme value of 218 mm in September 2012 also shows some underestimation in the simulation. Nevertheless, when comparing the monthly average runoff values, the three stations recorded 64 mm, 52 mm, and 53 mm, respectively, with corresponding simulated values of 65 mm, 45 mm, and 50 mm. The deviation rates are 1.6%, 13.4%, and 5.7%, respectively. Except for the slightly larger deviation in the simulated data at the Gaoping station, the other two stations accurately reflect the monthly surface runoff data and their variations.

In summary of the Validation phases, the overall GCM-Meteorological data-surface runoff model framework demonstrates, through its simulation results during the Validation period, that the data can effectively simulate the monthly averages of surface runoff. However, a few extreme values are difficult to fully reproduce, and the model can only indicate the presence of extreme conditions. Specifically, the simulated data show relatively high values, but there is still a gap compared to the observed maximum values. Since this data will be used for qualitative assessments of whether significant flood disasters have occurred, it can be considered as referential.

### Model prediction

**Fig. 5:**
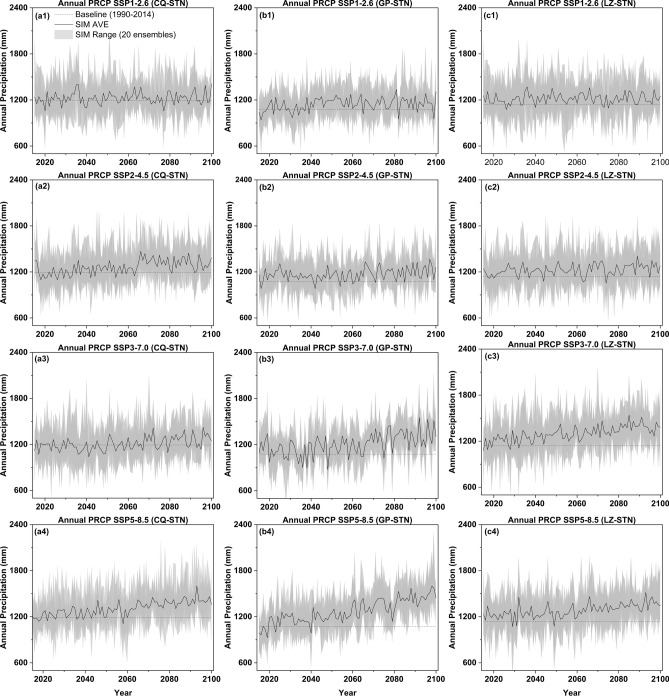
Prediction of simulated annual precipitation from 2015 to 2100 under four Shared Socio-economic Pathways (SSP1-2.6, SSP2-4.5, SSP3-7.0, and SSP5-8.5) at 3 stations: (**a**) Chongqing, (**b**) Gaoping, and (**c**) Luzhou Station.

Through model Validation, the entire research framework will proceed to simulate and predict future meteorological variables and surface runoff. The training data for this part of the model covers the period from 1990 to 2014, with predictions extending from 2015 to 2100. For the future economic scenarios, four different Shared Socioeconomic Pathways (SSPs) are employed: SSP1-2.6, SSP2-4.5, SSP3-7.0, and SSP5-8.5. Figure 5 presents the annual rainfall trends at three stations under various scenario projections for the future period (the model simulates monthly data, which has been aggregated into annual data for better illustration of inter-annual trends). The reference benchmark values for the three stations, based on the average from 1990 to 2014, are 1192 mm, 1076 mm, and 1141 mm, respectively. Under the SSP1-2.6 scenario, the annual precipitation at these three stations is projected to remain relatively stable in the future, as illustrated in Fig. 5a1, b1, and c1. The annual average precipitation at these stations is expected to peak by the mid-century, with the average annual precipitation for the period 2041–2070 reaching 1232 mm, 1171 mm, and 1231 mm, respectively. This represents an increase of 3.4%, 8.8%, and 7.9% compared to the reference benchmark values. Under the SSP2-4.5 scenario (Fig. 5a2, b2, c2), the annual precipitation changes exhibit some similarities with those under the SSP1-0.26 scenario, both being relatively gradual. However, the peak values occur at the end of this century (2071–2100), with the average values of annual precipitation at the three stations reaching 1319 mm, 1203 mm, and 1250 mm, respectively. Under both the SSP3-7.0 and SSP5-8.5 scenarios, annual precipitation exhibits a marked upward trend, with Gaoping showing a particularly pronounced increase. In the extreme SSP5-8.5 scenario, the average growth rates for the three stations by the end of this century reach 16.9%, 31.9%, and 17.8% respectively. Within this scenario, the increases in Chongqing and Luzhou are relatively more gradual, whereas Gaoping experiences more drastic changes. Especially during the mid-century period from 2055 to 2065, its simulated average precipitation annual data ranges from a minimum of 1117 mm to a maximum of 1440 mm, indicating a relatively unstable rainfall trend.

**Fig. 6:**
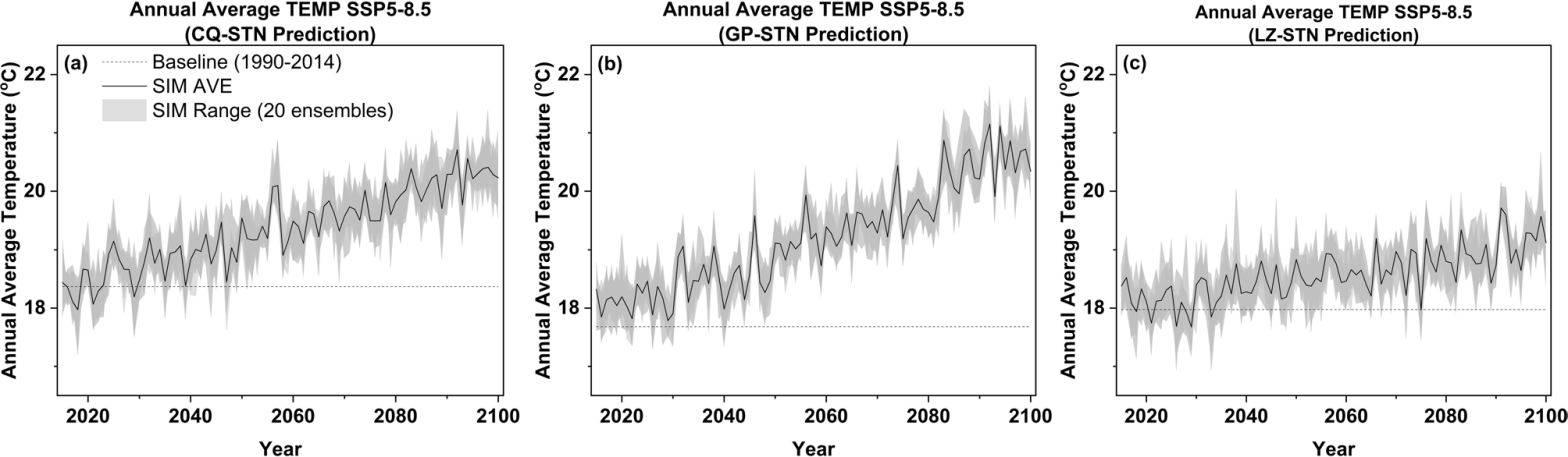
Prediction of simulated annual average temperature from 2015 to 2100 under SSP5-8.5) at 3 stations: (**a**) Chongqing, (**b**) Gaoping, and (**c**) Luzhou Station.

Figure 6 presents the predicted annual average temperature at three locations during the future period under SSP5-8.5 scenario. From the images, it can be observed that under the extreme shared socioeconomic pathway (SSP5-8.5), the annual mean temperatures in Chongqing and Gaoping exhibit a notably pronounced and consistent upward trend. In contrast, the projected data for the Luzhou station shows a more moderate trend compared to the other two sites. By the mid-century (2041–2070), the average temperatures at these three stations are expected to reach 19.3 °C, 19.1 °C, and 18.6 °C, respectively. Towards the end of the century (2071–2100), the average temperatures are projected to continue rising to 20.0 °C, 20.2 °C, and 19.2 °C, respectively. In terms of growth rate, Gaoping location is expected to experience the most significant increase of 14.2% compared to the baseline temperature by the end of the century, a pattern similar to that observed in annual precipitation. Taking into account the annual temperature deviation of 0.7 °C within the region during the baseline period (1990–2014), the final model predicts a maximum temperature difference of approximately 1.2 °C among the three locations by the end of this century, indicating an increasing trend in temperature disparities between stations. Additionally, the model highlights an issue: due to the considerable distance between the three locations and the model’s framework not accounting for spatial correlation, there may be some spatial bias in the predictions for the three stations. Further refinements can be made to the model in the future to address this.

This study also analyzed the statistical characteristics of daily rainfall, focusing particularly on changes in monthly rainfall amount and wet-day probability under climate change. The results are presented in Fig. S3 (Supplementary Materials).

**Fig. 7:**
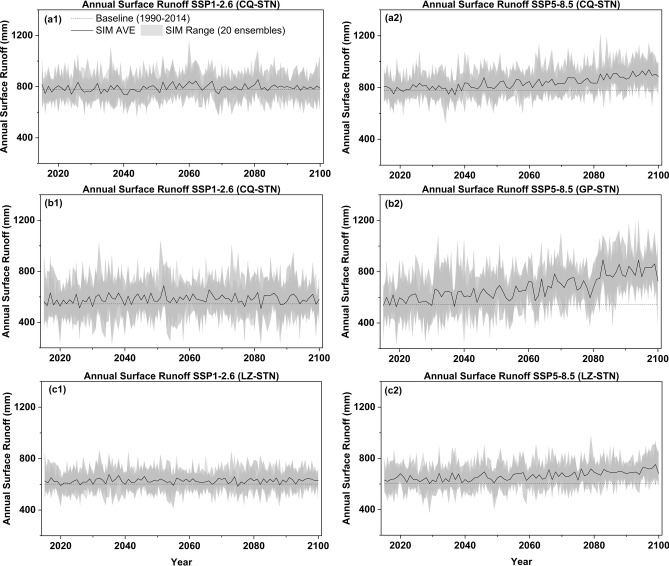
Prediction of annual surface runoff under SSP1-2.6 and SSP5-8.5 from 2015 to 2100 at three stations: (**a**) Chongqing Station; (**b**) Gaoping Station; (**c**) Luzhou Station.

Based on predicted meteorological variables (temperature and precipitation), this study forecast the surface runoff data under future climate change scenarios. Figure 7 illustrates the annual surface runoff variations under two shared socioeconomic pathways (SSP1-2.6 and SSP5-8.5). Under SSP126, the surface runoff at the three stations exhibits relatively stable changes, with peaks generally occurring in the mid-twenty-first century (2041–2070), reaching 796 mm, 591 mm, and 627 mm respectively. The corresponding growth rates compared to the baseline values are 2.5%, 8.6%, and 4.1%, respectively. In the SSP5-8.5 scenario, the three stations show a continuous increase, with annual runoff reaching 879 mm, 765 mm, and 692 mm by the end of the century (2071–2100), representing growth rates of 13.2%, 40.5%, and 15.0%, respectively. This pattern aligns closely with changes in precipitation, suggesting that Gaoping may face significant flood risk in the future.

### Flood risk identification

Table 2 compares the simulation performance of PCA-RF models trained with and without SMOTE preprocessing. The model was calibrated using data from 1990 to 2006 and validated against the 2007–2014 dataset. Observational records indicate that severe flood events occurred in only 5% of the total study months, revealing significant class imbalance in the original dataset. To address this imbalance, SMOTE was applied to the training dataset to equalize the representation of flood and non-flood months. Results demonstrate that the SMOTE-enhanced PCA-RF model outperformed its non-SMOTE counterpart in both accuracy and reliability. Specifically:

- Predictive accuracy: The SMOTE-processed model exhibited narrower interquartile ranges (IQRs) across 20 simulation trials (90.63%–93.75%) compared to the non-SMOTE model, indicating enhanced robustness.
- Event probability estimation: The SMOTE-integrated model achieved a mean probability density estimate of 4.56% for severe flood occurrence, closely aligning with the observed frequency of 4.2%. Its IQR (4.12%–4.89%) also showed reduced variability relative to the non-SMOTE approach.

**Table 2:** Comparative analysis of monthly probabilities of severe flood events performance between PCA-RF models with and without SMOTE using hydrological variables.

	Validation					
	Without SMOTE			SMOTE		
	Accuracy rate			Accuracy rate		
	TP25	Average	TP75	TP25	Average	TP75
OBS	89.84%	91.30%	92.71%	90.63%	91.67%	93.75%

	Incidence rate of disasters			Incidence rate of disasters		
	TP25	Average	TP75	TP25	Average	TP75
4.20%	3.13%	5.00%	6.25%	3.39%	4.56%	5.21%

**Fig. 8:**
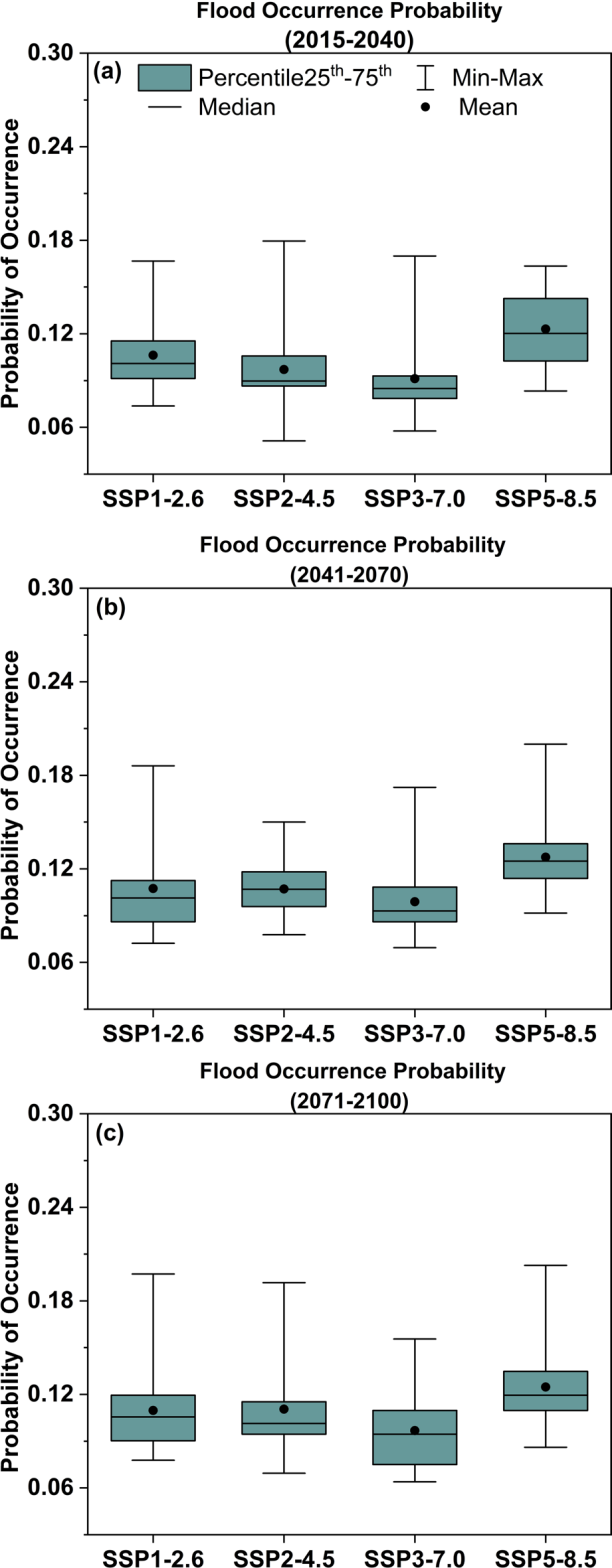
Projected decadal evolution of sever flood occurrence probability under climate change scenarios. (**a**) Near-term projection (2015–2040); (**b**) mid-century projection (2041–2070); (**c**) end-century projection (2071–2100). Scenario specifications: SSP1-2.6 (low radiative forcing), SSP2-4.5 (intermediate), SSP3-7.0 (high), SSP5-8.5 (very high).

These findings confirm that the SMOTE-optimized PCA-RF framework effectively mitigates class imbalance-induced biases while improving simulation stability. Consequently, this integrated methodology has been selected for future flood projections under different SSPs during the future period, and presented in Fig. 8.

- Near-term projections (Fig. 8a, 2015–2040)

Baseline simulations indicate monthly flood probabilities ranging from 9.12% (SSP3-7.0 scenario) to 12.31% (SSP5-8.5 scenario). The SSP3-7.0 ensemble demonstrates the lowest risk (mean = 9.12%, IQR: 7.78–9.29%), while SSP5-8.5 yields the highest probability (mean = 12.31%, peak = 14.34%), exceeding current observational baselines by > 200%.

(b) Mid-century projections (Fig. 8b, 2041–2070)

SSP1-2.6 and SSP2-4.5 scenarios show comparable mean probabilities (10.15% vs. 10.22%), though SSP2-4.5 exhibits narrower IQRs (9.87–10.61%) suggesting higher confidence in elevated risks. SSP3-7.0 maintains the lowest probability (9.89%, + 8.4% vs. 2015–2040), while SSP5-8.5 shows intensified risks (mean = 12.75%, IQR: 11.39–13.75%) with left-skewed distribution compared to earlier projections.

(c) End-century projections (Fig. 8c, 2071–2100)

SSP1-2.6 and SSP2-4.5 scenarios exhibit marginal probability increases (less than 1.2%). SSP3-7.0 displays expanded uncertainty (IQR width + 37%) despite a slight mean decrease to 9.68% (– 2.11% vs. mid-century). SSP5-8.5 projections stabilize at 12.71–12.83%, maintaining peak risk levels.

Collectively, these findings demonstrate that all SSPs demonstrate ascending flood probabilities over time, with SSP5-8.5 (incorporating extreme weather intensification and modified hydrological regimes) showing 2–3 times higher probabilities than historical baselines. SSP3-7.0 exhibits paradoxically moderated growth (+ 8.4% mid-century vs. + 15.2% in SSP2-4.5), potentially reflecting its socioeconomic assumptions about land-use regulation. This pathway divergence stems from the structural paradigm of SSP frameworks: SSP5-8.5 embodies a fossil-intensive development trajectory generating strong radiative forcing, whereas SSP3-7.0 reflects constrained climate adaptation allocations (less than 0.5% GDP) under regionally fragmented governance regimes. Their distinct land–atmosphere coupling processes drive fundamentally divergent hydrological responses.

## Conclusions and discussion

This study evaluates and analyzes the future flood risk in the Sichuan Basin, one of the regions most severely impacted by flood disasters in China, under the influence of climate change, by establishing a research framework system integrating GCM, meteorological variables, surface runoff, and flood risk assessment. Through analysis, Projections indicate that under extreme emission scenarios, the annual precipitation in the Sichuan Basin is expected to increase by approximately 17% to 32% by the end of this century. Concurrently, model simulations reveal three notable precipitation regime modifications: daily precipitation events are projected to become more temporally concentrated, rainfall intensity is expected to escalate significantly, and monthly rainfall distribution is anticipated to shift progressively toward the dry season. Furthermore, model analyses substantiate that the region is projected to manifest a warming trend of 1.0 °C to 2.5 °C in annual mean temperature. Consequently, the annual surface runoff in the study area is expected to increase by 13% to 40%. By classifying the aforementioned data using the PCA-RF model, it is predicted that the monthly probability of sever flood disasters in this region will more than double by the end of the century. Therefore, these findings necessitate implementing adaptive risk governance frameworks in the study area, with sustained monitoring of flood hazards and mitigation strategies extending beyond conventional flood season boundaries. Particularly, hydrological monitoring should prioritize emerging flood-prone windows identified in precipitation regime shifts under climate change scenarios.

This study presents the following key advances:

1. Development of a hybrid physics-informed machine learning framework for assessing the impacts of climate change on flood hazard frequency. The modular framework integrates distinct components for: spatial downscaling of climate variables, hydrological process modeling, temporal disaggregation, and extreme-value adjustment. Its modular design facilitates performance optimization, allowing individual components to be replaced with superior-performing algorithms to enhance overall framework accuracy and adaptability for rapid deployment to regions in need.
2. Incorporation of multi-source data (including observational datasets and reanalysis products), enhancing applicability in data-sparse regions. This capability contributes significantly to the goal of establishing comprehensive early warning systems, thereby strengthening disaster prevention and mitigation efforts.

Notably, this study has significant scope for future improvement:

1. Enhanced integration of physical mechanisms: While the current machine learning framework incorporates physical reasoning in variable selection and establishes statistical relationships between GCM outputs and local conditions, it inherently relies on the stationarity assumption of these relationships for future projections. Future research should prioritize hybrid approaches, such as using machine learning to optimize parameters within physics-based models, thereby strengthening the representation of physical processes and improving predictive accuracy.
2. High-temporal-resolution flood prediction: This study focused on [mention the temporal scale used, e.g., daily/monthly] runoff simulations. Quantitative prediction of flood magnitudes at finer temporal resolutions (e.g., sub-daily or hourly scales) is essential for detailed flood forecasting and risk assessment and represents a critical direction for future work.

In summary, under the constraint of data scarcity, this study innovatively applies a research framework and method for flood risk assessment to the Sichuan Basin. The framework and method have been validated and provide predictions for the future occurrence probability of floods, thus offering a basis for future disaster prevention and mitigation efforts.

## Supplementary Information

Supplementary Information.

## Data Availability

The datasets generated during this study are available from the corresponding author upon reasonable request, subject to compliance with institutional data access protocols and approval by the relevant ethics committee.
