# Supplementary material for: A multi-source data-driven framework for probabilistic flood risk assessment using cascade machine learning models: case study in the Sichuan Basin
Source: Sci Rep. 2025 Jul 23;15:26706. doi: 10.1038/s41598-025-12391-y (PMC12284062; doi:10.1038/s41598-025-12391-y)
Supplement: Supplementary file 1 — Supplementary Information. [file 41598_2025_12391_MOESM1_ESM.docx]

**Supplementary Materials**


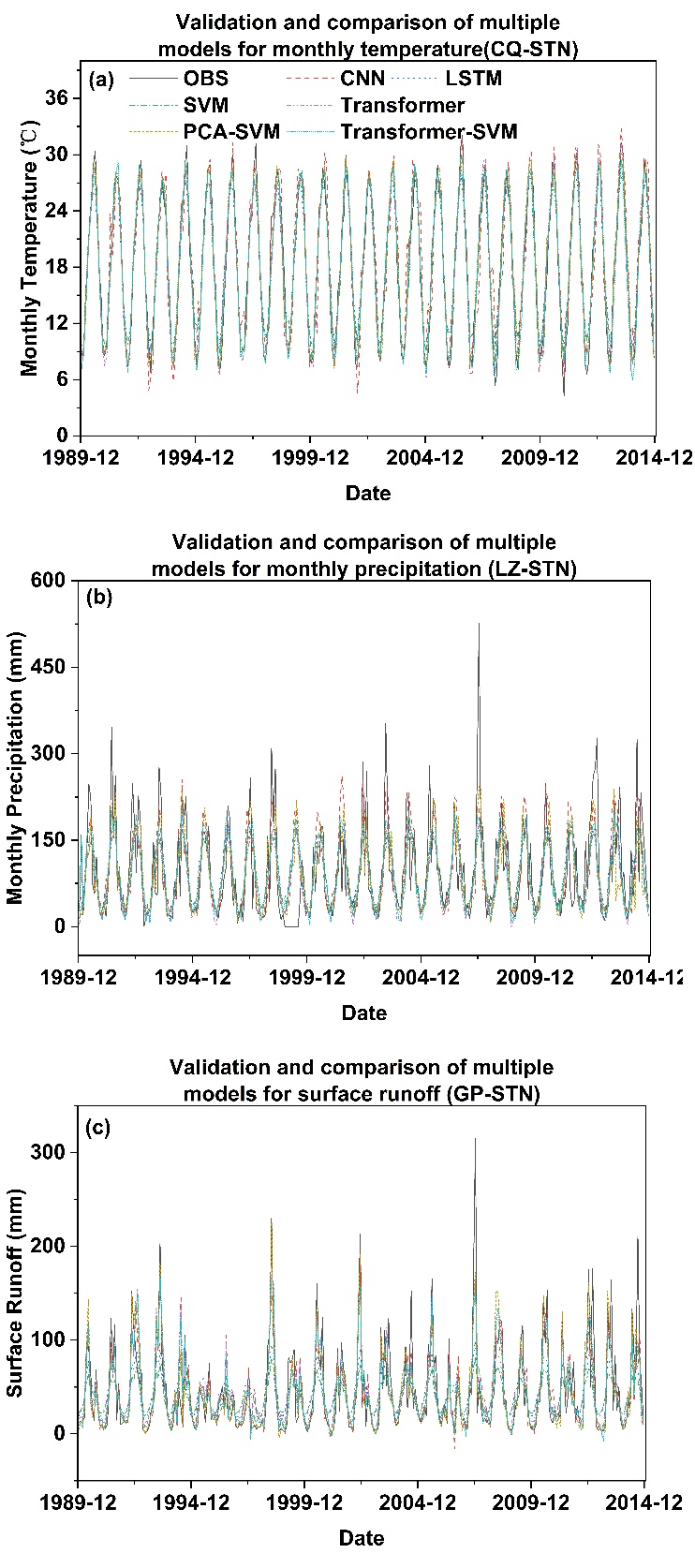


**Fig. S1.** Validation and comparison of multiple models in simulating (a) monthly temperature at CQ-STN, (b) monthly precipitation at LZ-STN, and (c) surface runoff at GP-STN during 1990-2014.

To validate the performance of the machine learning models employed in the three distinct components of the proposed atmosphere-flood probabilistic risk framework – namely, temperature downscaling, precipitation downscaling, and surface runoff modeling – this section utilizes observational data as input (training period 1990-2006, validation period 2007-2014). Unlike the main text where surface runoff simulation relies on outputs from the downscaling stage, this specific validation contrasts the component-specific models against traditional standalone models (including CNN, LSTM, SVM, and Transformer). The objective is to ensure that the most suitable model is selected for each component, thereby mitigating the propagation and accumulation of bias throughout the overall modeling framework.

**Fig. S1(a)** presents the temperature downscaling performance at the CQ-STN station. All models demonstrated high accuracy, with correlation coefficients (R) exceeding 0.946. The Transformer and Transformer-SVM models achieved the highest R values (0.982 and 0.980, respectively). However, considering the RMSE, Transformer-SVM slightly outperformed Transformer (1.511°C vs. 1.533°C). Therefore, Transformer-SVM was selected for the temperature downscaling component.

**Fig. S1(b)** shows precipitation downscaling results at the LZ-STN station. Due to the higher stochasticity and weaker correlation with GCM variables compared to temperature, model performance was generally lower, particularly in simulating extreme values (further discussed and addressed in the Methodology section). Transformer-SVM and LSTM yielded the lowest RMSE values (63.412 mm and 64.509 mm, respectively), while other models exceeded 65 mm. Consequently, Transformer-SVM was also chosen for the precipitation downscaling component.

**Fig. S1(c)** depicts the surface runoff simulation performance. Based on the NSE, Transformer, Transformer-SVM, and PCA-SVM exhibited comparable results, with Transformer-SVM achieving the highest NSE (0.690) and PCA-SVM the lowest among these three (0.684). However, analysis of the PBIAS revealed that PCA-SVM performed significantly better (-12.317% vs. -13.122% for Transformer and -39.688% for Transformer-SVM). All models underestimated observations, especially extremes, but PCA-SVM exhibited the lowest bias. Therefore, PCA-SVM was selected for surface runoff modeling. Notably, model performance at the CQ-STN station was generally lower (NSE < 0.6), with PCA-SVM achieving an NSE of only 0.560. Though still acceptable, this highlights the need for further model improvement, as discussed later.

Overall, the models selected for each component within the atmosphere-flood probabilistic risk framework (e.g., Transformer-SVM for downscaling, PCA-SVM for runoff) demonstrated relatively strong performance. However, their superiority over alternative models was marginal. Consequently, the framework is designed to accommodate future substitution of individual components with superior-performing models, thereby enhancing overall performance. This modular approach constitutes a key aspect of the framework's flexibility.


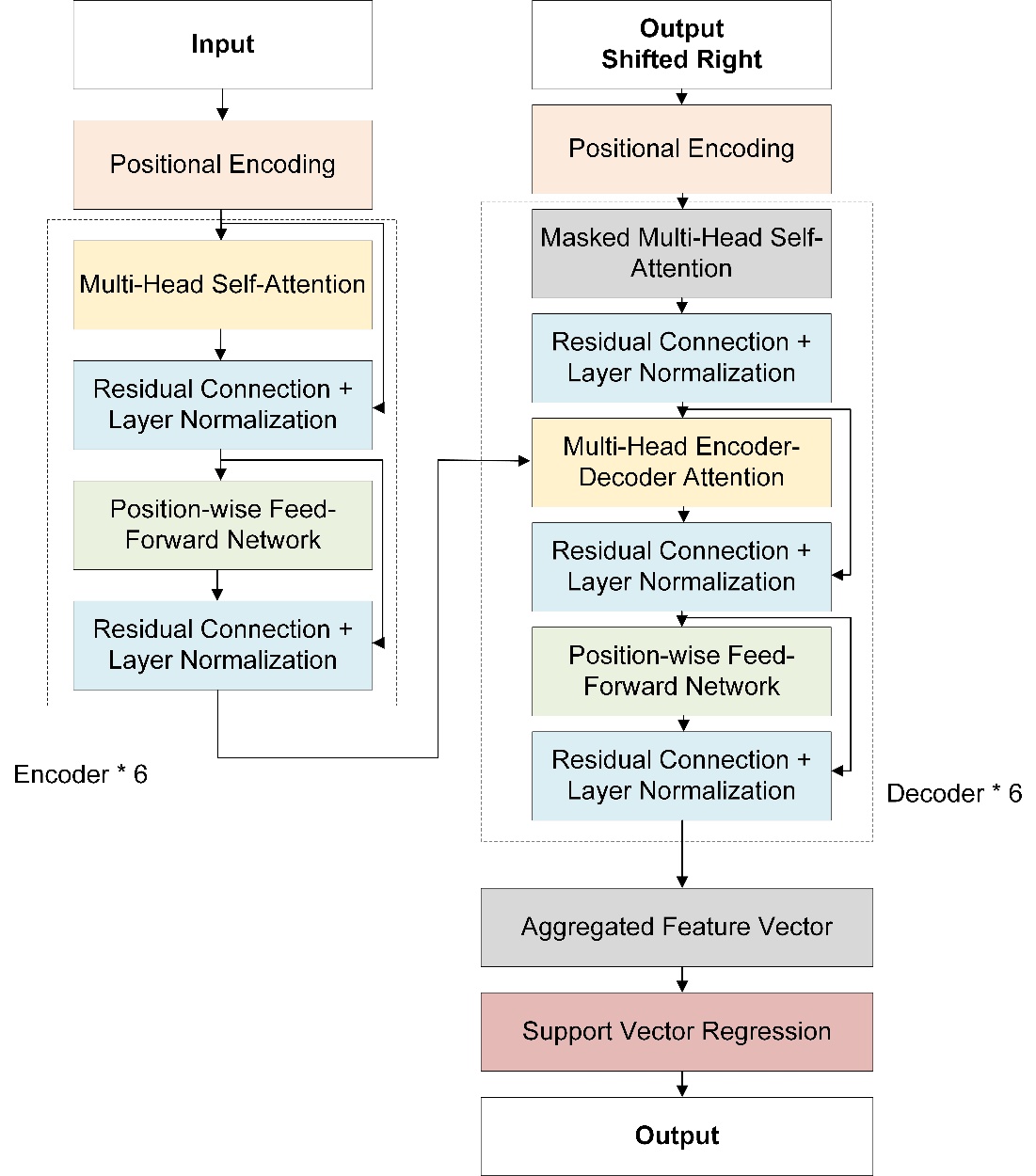


**Fig. S2.** Structure of Transformer-SVM model.

**Fig. S2** illustrates the basic architecture of the Transformer-SVM model. The model employs a serial coupling between the Transformer and SVM components. Specifically, the Transformer module utilizes an encoder-decoder structure with attention mechanisms to extract integrated feature vectors from the input data. These feature vectors are subsequently fed into the SVM for regression prediction.


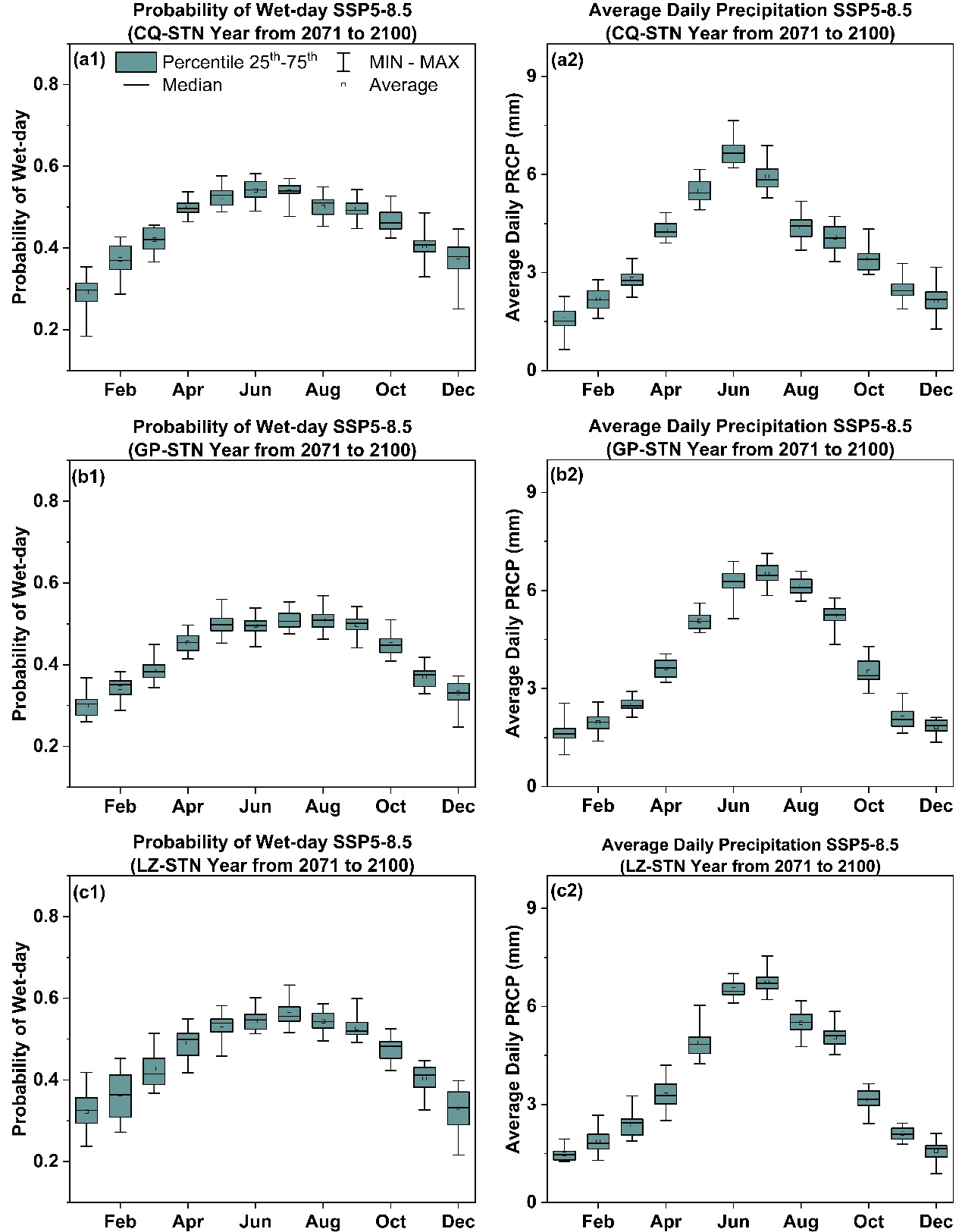


**Fig. S3.** Prediction of statistical characteristics Probability of Wet-day and Average daily precipitation per month under SSP5-8.5 during the end of this century from 2071 to 2100 at three stations: (a) Chongqing Station; (b) Gaoping Station; (c) Luzhou Station.

**Fig. S3** presents the prediction of two statistical characteristics, the probability of wet days (**Figure S3 a1**, **b1** and **c1**) and the average daily precipitation per month ((**Fig. S3 a 2**, **b2** and **c2**) under the SSP5-8.5 scenario, spanning from 2071 to 2100 at the end of this century at three locations. Firstly, **Fig. S3 a1**, **b1**, and **c1** illustrate that the average probability of wet days for the three stations at the end of this century is 45.2%, 42.9%, and 46.0% respectively, with corresponding baseline observational values of 45.3%, 40.2%, and 51.1%. This indicates that the annual number of rainy days remains relatively stable at the Chongqing and Gaoping stations, while there is a slight upward trend at the Luzhou station. Meanwhile, when combined with the 16%-31% increase in annual precipitation observed at these three locations during this period, it becomes evident that the rainfall intensity has shown a significant increase across all three sites, particularly at Luzhou. From **Fig.S3 a2**, **b2**, and **c2**, we can observe the trends in daily average rainfall at the three stations, particularly during the traditional dry season from November to February of the following year. Notably, in February, the daily average rainfall growth rates at the three stations reached 160.3%, 159.6%, and 79.5%, respectively. However, the average rainfall percentages in February increased by 32.3%, 8.4%, and decreased by 9.1% respectively, suggesting that February could become a month prone to frequent flood disasters towards the end of this century. In conclusion, the diurnal precipitation regime in the study area is projected to undergo substantial transformations by the century's end (year 2071-2100). Under the SSP5-8.5 scenario, daily precipitation will demonstrate intensified concentration patterns with amplified rainfall intensity. Concurrently, a significant temporal redistribution of monthly precipitation is anticipated, particularly revealing unexpected rainfall increments during historically arid intervals, as exemplified by February's conventional dry season."
